# Nanoporous materials with predicted zeolite topologies†

**DOI:** 10.1039/d0ra01888k

**Published:** 2020-05-08

**Authors:** Vladislav A. Blatov, Olga A. Blatova, Frits Daeyaert, Michael W. Deem

**Affiliations:** Samara Center for Theoretical Materials Science (SCTMS), Samara University Ac. Pavlov St. 1 443011 Samara Russian Federation blatov@topospro.com; Samara Center for Theoretical Materials Science (SCTMS), Samara State Technical University Molodogvardeyskaya St. 244 443100 Samara Russian Federation; Department of Bioengineering, Rice University 6100 Main St Houston TX USA mwdeem@rice.edu; FD Computing Stijn Streuvelsstraat 64 2340 Beerse Belgium; Department of Physics & Astronomy, Rice University 6100 Main St Houston TX USA

## Abstract

An increasing number of newly synthesized materials have been found to be previously present in databases of predicted porous materials. This has been observed not only for zeolites, but also for other inorganic materials and for MOFs. We here quantify the number of synthesized zeolites that are present in a large database of predicted zeolite structures as well as the number of other inorganic crystals and MOFs present in this same database. We find a significant number of real materials are in this predicted database of zeolite-like structures. These results suggest that many other predicted structures in this database may be suitable targets for designer materials synthesis.

## Abstract 2

Topological exploration of crystal structures demonstrates the presence of known zeolites, inorganics, and MOFs in a database of predicted materials.

## Graphical abstract

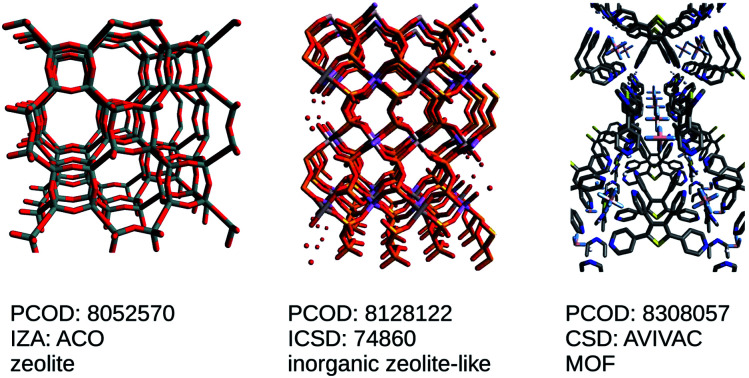

## Introduction

Zeolites are nanoporous crystal forms of aluminosilicate oxides that are widely used in catalysis and adsorption.^1^ At present, 248 zeolite topologies are known.^2^ These structures differ in the connectivity and relative ordering of the TO_4_ (T = Si or Al) tetrahedra, and therefore in the size and shape of the nanoporous cavities. In addition to aluminosilicate zeolites, numerous materials with zeolite topologies but containing other elements or building blocks that serve as tetrahedral centers and bridging atoms are known.^3,4^

Due to their importance in many industrial processes, the discovery and identification of novel zeolite and zeolite-like materials is a field of intensive research.^4^ To aid the search for new zeolites, computational methods have been applied to generate novel predicted topologies that expand upon the currently known materials.^5–7^ The PCOD database^8^ developed in the Deem laboratory contains predicted zeolite structures that have both a low computed energy gap with respect to alpha-quartz, and an energy/density ratio that is in the range of known zeolites. A considerable number of existing zeolites were found in the structures predicted during the generation of the database.^6^

The PCOD has been extensively screened in the search for zeolites with specific functionalities.^9–15^ The ToposPro program package^16^ is a computer program for the topological analysis of crystal structures allowing an objective description and comparison of crystal structures. Recently, the PCOD database has been made searchable with the ToposPro package and the corresponding online TopCryst service.^17^ This allows the comparison of newly discovered zeolite frameworks with the predicted frameworks in the PCOD. Thus, a number of recent candidates for the IZA database of known zeolites have been found to correspond to entries in the PCOD.^18–20^ In total, 154 of the 248 known zeolite frameworks in IZA were identified in the PCOD. The concept of structure representation in the ToposPro method also allows the comparison of topologies of different classes of materials such as inorganic compounds and coordination polymers. By searching the Inorganic Crystal Structure Database (ICOD^21^) and the Cambridge Structural Database (CSD^22^) we additionally found a large number of topologies that were predicted in the PCOD. Of these, 57 were zeolite-like inorganic materials, and 118 were metal–organic frameworks (MOF), another class of nanoporous materials.

Thus, a large number of predicted zeolite topologies present in the PCOD correspond to synthesizable compounds, be it zeolites or other nanoporous materials. This, in combination with the very large size and topological diversity of the database, is an incentive for further search efforts of this database in the design of novel materials with tailored properties.

## Methods

At the origin of the PCOD is a Monte-Carlo based algorithm to generate predicted zeolite-like frameworks by sampling and optimizing a zeolite figure of merit.^23^ Initially, approximately one million structures were found belonging to a limited number of space groups. The database was extended to include topologies of all space groups,^6^ and was refined by performing energy minimization with two force fields, SLC^25^ and BKS,^26^ using the GULP program.^27^ Of the thus obtained 2.7 million topologically unique, energy minimized structures, 313 565 were no higher in energy than 30 kJ (mol Si)^−1^ relative to quartz using the SLC force field, and 585 139 were no higher in energy than 65 kJ (mol Si)^−1^ relative to quartz using the BKS force field.^7^ These criteria are judged to be the limits for thermodynamically stable aluminosilicate zeolites, and therefore only these structures were retained in the database.

**Fig. 1:**
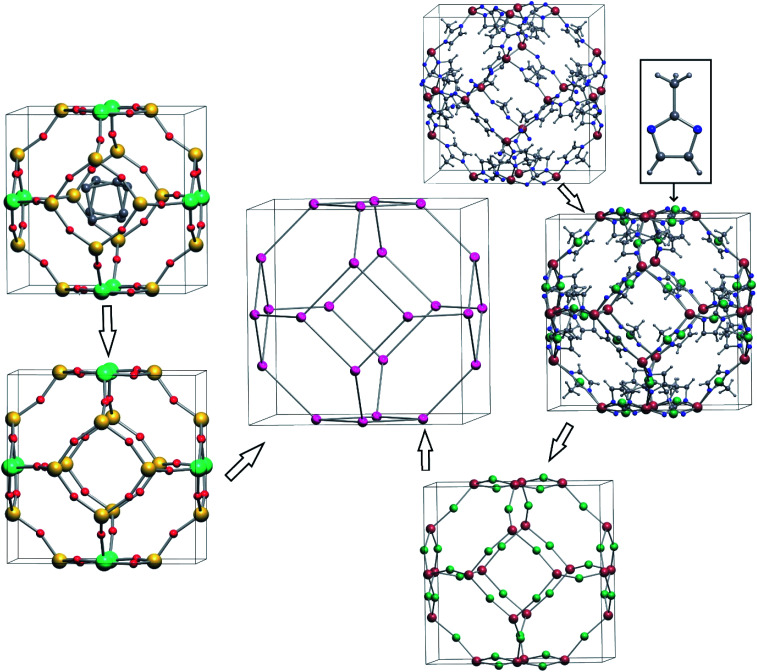
Simplification of a zeolite (left) and a MOF (right) structures to the zeolite framework, in this case sodalite (SOD). At the first step, all extraframework species are removed, and organic ligands are squeezed into their centers of mass. At the second step, all bridge nodes are transformed to the network edges. Aluminosilicate AlSi_5_O_12_ with unidentified extraframework organic species^31^ and [(methylimidazolato)_2_Zn]^32^ are the original zeolite and MOF structures in this example.

The ToposPro program package offers an objective and complete approach to explore crystal structures by analyzing their topology. In addition to coordination sequences, ToposPro computes the so-called point symbols and vertex symbols that collect the shortest cycles and rings (cycles without shortcuts) of atoms, respectively.^28^ The general scheme of the analysis includes the following steps:^29^ (i) determination of all interatomic interactions in the structure using a number of chemical and geometrical criteria; (ii) search for structural groups (building blocks) with unique topological algorithms; (iii) simplification of the structure by squeezing the structural groups into their centers of mass keeping the connectivity between the groups; (iv) determination of the topology for the resulting underlying net, *i.e.* the net of the centers of the structural groups, by comparison of the topological indices (coordination sequences, point and vertex symbols) of the underlying net with the indices for the reference topologies from the ToposPro TTD Collection. All these steps are performed in an automated mode, so thousands of crystal structures can be processed in an appropriate time. We have applied this procedure to determine the PCOD topologies and to identify which IZA zeolite topologies are present in the PCOD. Additionally, the Cambridge Structural Database (CSD, version 5.40 as of November 2018) and the Inorganic Crystal structure Database (ICSD, release 2019/2) were screened for PCOD topologies. Two classes of materials were distinguished: zeolite-like inorganic materials, and MOFs. For identifying the MOFs, the MOF building blocks were treated as T-centers, and the organic linkers as the oxygen atoms in the corresponding predicted zeolite structures. All frameworks (PCOD structures, inorganic materials and MOFs) were simplified to their underlying nets consisting of only T centers by replacing the linker nodes (L) by edges between the T centers, *i.e.* by the graph transformation T – L – T → T – T (Fig. 1). To designate the underlying topologies, besides the IZA symbols for zeolites, we use the RCSR three-letter symbols,^30^ the ToposPro *N*D*n* nomenclature, the Epinet *sqc* symbols, and Fischer's symbols *k*/*m*/*fn* for three-periodic sphere packings.^29^ For the PCOD topologies that have not been found in other resources, we use the ToposPro *N*D*n* symbols with the suffix HZ; for example, the ToposPro symbol 4,4T1319-HZ means that this is a predicted zeolite with two topologically inequivalent T nodes (*N* = 4,4), three-periodic framework (D = T) and the ordinal number *n* = 1319 among other topologically different predicted zeolites with two crystallographically distinct T nodes.

## Results

A total of 154 of the 248 IZA structures were found to be present in the PCOD. Additionally, by screening the CSD and ICSD, 72 zeolite-like and other inorganic materials and 118 MOFs were found to have topologies present in the PCOD.

Table SI1† lists the 154 PCOD structures that were identified in the IZA database. Column one provides the PCOD code, and column two provides the IZA code of each framework.

Table 1 lists the 72 zeolite-like and other inorganic structures. The first two columns provide the PCOD number and the number of actual structures found. Not all topologies are listed, but only those that are chemically close to zeolites. For each topology, the ICSD reference code and atomic composition of a selected structure are provided in columns three and four. The last column provides the symbol of the topology according to one of the nomenclatures described above.

**Table 1:** Inorganic zeolite-like materials from the ICSD with topologies present in the PCOD. IZA zeolites are excluded from this table. The entries with a **bold** PCOD number are inorganic compounds whose composition precludes their classification as zeolite materials, but which nonetheless have a zeolite-like topology of the listed PCOD number

PCOD	Number of structures	ICSD ref. code (example)	Formula (example)	Underlying topology
PCOD8000022	364	9829	NaAlSi_3_O_8_	**fel**
PCOD8322222	352	1109	SiO_2_	**lon**
**PCOD8249897**	274	33765	Na(FeO_2_)	**dia**
PCOD8149775	202	237135	Al_4_Fe_2_Si_5_O_18_	**brl**
PCOD8322767	97	23371	Na_2_Mg_5_Si_12_O_30_	4,4T45
PCOD8029344	79	87538	Na_4_(Al_3_Si_9_O_24_)Cl	**sca**
PCOD8167638	57	170497	SiO_2_	**pcl**
**PCOD8128122**	52	74860	LiAl(PO_3_)_4_	**mog**
**PCOD8046833**	49	20208	Li_3_(PO_4_)	*deh2*
PCOD8147992	46	18112	SiO_2_	**coe**
**PCOD8171653**	17	180997	Ca(Al_2_O_4_)	**byl**
**PCOD8128676**	13	74808	KCo(PO_4_)	**tpd**
**PCOD8033784**	10	246132	Li_2_Fe(SiO_4_)	4,4,4,4T130
**PCOD8249812**	9	41661	CaAl_2_O_4_	**cag**
PCOD8157925	9	68772	K(AlSi_2_O_6_)	**kea**
**PCOD8308057**	5	195287	Au(PO_4_)	**pts**
PCOD8152484	4	156657	BaNa_2_(AlSiO_4_)_4_	**bnl**
**PCOD8129062**	4	191544	Li_2_Mn(SiO_4_)	**sie**
**PCOD8128656**	3	171001	BaFe_2_O_4_	**baf**
**PCOD8321582**	3	151369	Ca_3_(Al_2_O_6_)	**nbo**
PCOD8170506	3	75659	SiO_2_	**irl**
**PCOD8045579**	2	33279	Na_2_Li_3_(FeO_4_)	*sqc*8104
PCOD8302308	2	170516	SiO_2_	**tzs**
**PCOD8000219**	2	173216	Li_3_(VO_4_)(H_2_O)_6_	**afw**
**PCOD8046854**	2	380488	KBSi_2_O_6_	4,4,4T91
**PCOD8129307**	1	1291	Be(PO_3_)_2_	4,4,4T159
PCOD8048022	1	56684	SiO_2_	4,4T39
**PCOD8154928**	1	60069	LiK(PO_3_)_2_	**zsn**
PCOD8255081	1	62584	SiO_2_	4,4,4,4T15
PCOD8166122	1	62585	SiO_2_	4,4,4,4T14
**PCOD8325712**	1	63414	Na_2.67_K_1.33_Zn_4_(PO_4_)_4_	4,4,4T148
PCOD8000277	1	75653	SiO_2_	**unc**
PCOD8128689	1	75664	SiO_2_	**bbe**
**PCOD8000217**	1	79705	K_2_(ZnSi_2_O_6_)	**bbi**
**PCOD8037080**	1	79705	K_2_(ZnSi_2_O_6_)	4,4,4,4,4T4
**PCOD8128125**	1	83427	Na_2_Zn(Si_2_O_6_)	**bbm**
PCOD8189369	1	83861	SiO_2_	4,4,4,4,4T3
PCOD8323349	1	89700	SiO_2_	**bam**
PCOD8000118	1	91681	Al(PO_4_)	**bcq**
PCOD8163521	1	92721	(Mg_0.917_Fe_0.083_)_2_Na_0.084_(Al_3.970_Fe_0.038_Si_4.992_)O_18_(H_2_O)_0.38_(CO_2_)_0.192_	**mot-e**
PCOD8307680	1	170479	SiO_2_	**wse**
PCOD8123215	1	170480	SiO_2_	**dei**
PCOD8321616	1	170493	SiO_2_	**umk**
PCOD8009546	1	170498	SiO_2_	**umq**
PCOD8170966	1	170499	SiO_2_	**umi**
PCOD8330894	1	170512	SiO_2_	**cbo**
PCOD8308285	1	170526	SiO_2_	**uot**
PCOD8308073	1	170528	SiO_2_	**uox**
PCOD8169643	1	170534	SiO_2_	**ukb**
PCOD8301974	1	170541	SiO_2_	4/5/*t*1
PCOD8297080	1	170543	SiO_2_	**lcs**
PCOD8129487	1	170545	SiO_2_	**gsi**
PCOD8123200	1	170546	SiO_2_	**mmt**
PCOD8045573	1	170547	SiO_2_	**neb**
PCOD8264888	1	173625	Al_15.68_(Si_41.1_Al_6.9_)O_96_	4,4,4,4T127
**PCOD8003420**	1	170960	Rb_4_(UO_2_)_2_(Si_8_O_20_)	4,4,4,4,4T7
PCOD8308378	1	261103	K_3.33_(C_2_H_5_)C_1.5_(Be_2_Si_12_O_33.7_)	**ksx**
**PCOD8328203**	1	92822	NaB(SiO_4_)	4,4,4T24-CA
**PCOD8185681**	8	167183	Zn(SiO_3_)	4,4,4T5318-HZ
**PCOD8120181**	4	4362	NaK(CuSi_4_O_10_)	4,4,4T4043-HZ
PCOD8170348	2	162489	K(AlSiO_4_)	4,4,4T5003-HZ
**PCOD8124771**	2	410869	K(ZnBP_2_O_8_)	4,4T1080-HZ
PCOD8283381	2	2116	Na_6_FeSi_8_Al_4_O_26_	4,4,4T6294-HZ
PCOD8127150	1	20797	K_2_BeSi_4_O_10_	4,4,4,4,4T243508-HZ
PCOD8052206	1	33924	H(AlSi_4_O_10_)	4,4T1048-HZ
**PCOD8308516**	1	59846	Zn(PO_3_)_2_	4,4,4T6862-HZ
**PCOD8321753**	1	65475	Li_3_K_3_(P_6_O_18_)(H_2_O)	4,4T1321-HZ
PCOD8099926	1	85474	Si_56_O_112_	4,4,4,4,4,4,4T28511-HZ
**PCOD8321730**	1	85734	Li_6_(P_6_O_18_)(H_2_O)_3_	4,4T1319-HZ
PCOD8071670	1	86548	SiO_2_	4,4,4,4,4,4T6819-HZ
PCOD8308593	1	86549	SiO_2_	4,4,4T6867-HZ
**PCOD8168418**	1	411142	Na_0.75_(NH_4_)_0.25_Zn(PO_4_)	4,4T1145-HZ

Table 2 lists the 118 MOF topologies. The first column provides the PCOD identifier and the second column the topology symbol. The bold entries have IZA zeolite topologies, the codes of which are listed in the third column. Column four lists the total number of structures found in the CSD, and the last column provides the CSD code of one of these structures as an example.

**Table 2:** MOF topologies from the CSD found in the PCOD. The entries with a **bold** PCOD number are also known IZA zeolite topologies

PCOD code	Underlying topology	IZA code	Number of structures	CSD ref. code (example)
PCOD8249897	**dia**		2291	XEYXUW
PCOD8308057	**pts**		632	AVIVAC
PCOD8128122	**mog**		299	PUZBES
PCOD8321582	**nbo**		258	TANNUU
**PCOD8321332**	**sod**	SOD	186	XIZDER
**PCOD8067826**	**crb**	BCT	120	PUMNIV
**PCOD8000282**	**gis**	GIS	96	DIZJED
PCOD8322222	**lon**		85	FIPXAF
PCOD8171811	**bbf**		70	QAVDEW
PCOD8249812	**cag**		51	KOTPUG
PCOD8000277	**unc**		41	VAHWOS02
PCOD8045573	**neb**		40	ANUPIK
**PCOD8162585**	**dft**	DFT	22	HIFVOI
PCOD8297080	**lcs**		22	GIZJUV
**PCOD8306957**	**rho**	RHO	20	MECWOH
PCOD8321454	**uni**		20	DIVPUU
**PCOD8077978**	**gme**	GME	16	RIRDAZ
PCOD8164109	**frl**		15	VEPBOK
PCOD8170506	**irl**		14	DEXXOU
PCOD8000219	**afw**		11	DAGFUP
**PCOD8068050**	**cha**	CHA	11	NIRKAB
**PCOD8238986**		THO	10	BEFNAD
PCOD8123200	**mmt**		10	DUWREU
**PCOD8308045**	**ana**	ANA	10	GUPDOL
**PCOD8306691**	**mer**	MER	8	EWENUR
**PCOD8170814**	**npo**	NPO	7	SODKIH
**PCOD8308796**		SAV	6	LOFZUB
**PCOD8308791**	**edi**	EDI	6	XAQTOY01
**PCOD8117704**		LAU	5	YOMVIG
PCOD8123215	**dei**		5	TOBQAE01
**PCOD8115801**	**ast**	AST	5	IRUROC
**PCOD8001707**	**can**	CAN	5	PAJSAX
**PCOD8307996**	**fau**	FAU	5	XEQNIQ
**PCOD8304448**	**mtn**	MTN	4	GAQYIH
PCOD8160106	4,4T67		4	QUDKIK
**PCOD8122541**		OWE	4	BEFNOR
**PCOD8307029**	**asv**	ASV	4	GOMSUW
PCOD8324721	**unj**		4	UFAQIE
PCOD8129487	**gsi**		4	ZUYWAR
**PCOD8077973**	**cgs**	CGS	3	DEPTOH
PCOD8077922	**cfc**		3	XACFAJ
PCOD8227613	**bbh**		3	ADECEU
PCOD8123876	4,4,4,4,4,4T10		3	EXOKIM
**PCOD8052570**	**pcb**	ACO	3	DEJROB
PCOD8055858	4,4T133		3	TAXHUX
PCOD8163960	4,4T85		3	WUPTIM
PCOD8163521	**mot**-e		3	FIWJIG
**PCOD8125027**	4,4,4,4,4,4,4,4T11	JNT	3	SOQJIT01
**PCOD8095118**	**lev**	LEV	3	TOFWEQ
PCOD8331046	**sdt**		2	ALIBUT
PCOD8330894	**cbo**		2	DOLWEI
PCOD8047042	**noq**		2	LATCIS
PCOD8126401	4,4,4,4T11		2	AMBZAG10
**PCOD8085224**	**sas**	SAS	2	VAHSIH
PCOD8045484	4,4T10		2	BOSCET
**PCOD8321918**	**afx**	AFX	2	OSUSAY
**PCOD8156657**		JRY	2	MORZID
PCOD8167638	**pcl**		2	RIDKOE
**PCOD8117232**		JSN	2	DARJOX
**PCOD8295280**		SAF	2	SUSZIQ
**PCOD8248916**		JSW	2	HATSEC
**PCOD8077977**		AEI	2	BEFPAF
PCOD8128125	**bbm**		2	MUNQIX
**PCOD8124791**	**ucn**	SBN	2	FIGQIV
PCOD8323892	4,4T148		2	RIRDED
**PCOD8169309**	**bik**	BIK	2	YOMBOS
PCOD8185531	**bbg**		1	MUDHOK
PCOD8171792	**cdp**		1	ZAYFEN
PCOD8217418	**stc**-4,4-Ccce		1	PUWQAA
PCOD8047071	4,4,4T162		1	OKUWOI
PCOD8123892P	**fsg**-4,4-Cmmm		1	XUNTEH01
PCOD8156062	**cus**		1	XUNSOQ
**PCOD8324445**	**afi**	AFI	1	IMIDZB13
PCOD8255216	4,4,4T206		1	KALXUT
PCOD8041061	**ukn**		1	OBAWOG
PCOD8308449	**kat1**		1	OFERUN08
**PCOD8056793**	**phi**	PHI	1	BEFMAC
**PCOD8307701**	**kfi**	KFI	1	JILWOR
PCOD8129205	4,4T101		1	PAPHOF
PCOD8047025	4,4,4T33		1	MUPLAL
**PCOD8324260**		SFW	1	OSUSIG
PCOD8000235	4,4T146		1	NIJTUX
PCOD8146884	**Sqc**973		1	DOKJIX
**PCOD8054148**	**afy**	AFY	1	COQNIF
PCOD8302308	**tzs**		1	OXEVOE
PCOD8125166	4,4,4T43		1	DOHBAE
PCOD8125020	4,4,4,4,4,4,4,4T18		1	SUWZUH
**PCOD8324829**		MEI	1	YUTFAW
**PCOD8078892**		USI	1	IJIGOX
PCOD8118604	4,4T16		1	TOQBUW
PCOD8126974	4,4,4,4T72		1	SOCJUR
PCOD8111377	4,4T168		1	QUMJAL
PCOD8129307	4,4,4T159		1	MURFEM
PCOD8054312	4,4T21		1	GIMWAB
**PCOD8095768**	**sat**	SAT	1	PAQJUM
PCOD8305504	4,4,4T60		1	HABREJ
PCOD8308885	4,4T131		1	QUBWIU
PCOD8308885	**zec**		1	HICGEG
PCOD8129062	**sie**		1	BEFLUW
**PCOD8123580**		ZON	1	NETRIN
PCOD8121794	**sqc**3848		1	CODSOF
PCOD8111380	4,4T23		1	ICIZAV
PCOD8128437	**sta**-4,4-Cccm		1	EMAYUM
PCOD8122913	4,4T255		1	VALVEM
**PCOD8125830**		AFN	1	AXUPEO
PCOD8134958	4,4,4T68		1	LUZZEM
PCOD8187865	**itv**		1	GUPCUQ02
PCOD8047418	4,4T46		1	BOQTEI
PCOD8187185	**umr**		1	SAZPOB
PCOD8056515	**ntn**		1	USOXIL
**PCOD8011377**	**jbw**	JBW	1	IGUCIX
PCOD8171653	**byl**		1	GUKLOO
PCOD8308073	**uox**		1	WEMWAP
PCOD8136892	4,4,4,4T5		1	NISPEL
PCOD8014403	4,4,4,4,4,4T1		1	IGEXUN
PCOD8036144	4,4,4T81		1	QUPHOZ
**PCOD8228636**	**att**	ATT	1	FECCIZ
**PCOD8076973**		BOF	1	BAXMUI

Table 3 lists 11 IZA topologies not found in the PCOD database, but present in MOFs. Also listed are the two interrupted structures not eligible for inclusion in the PCOD database. The first column provides the IZA code of each framework. The second column provides the number of actual structures found. The third column provides the ICSD reference code.

**Table 3:** MOFs from the CSD, which possess IZA zeolite topologies, but which are not contained in the PCOD

IZA code	Number of structures	CSD ref. code (example)
ABW	397	LABPIP
ATN	8	EYUKOZ
BSV	7	XUWTEO
CGF	1	NIVRAL
-CLO	1	ZAZNUL
CZP	2	XUWSUD
DFO	2	SIHFAQ
-LIT	1	GADWAL
LTA	8	HITYEP
PUN	1	RUMXUT
RWY	10	MUNBAY
SOS	2	MANKIW
WEI	3	FAHQEN

## Discussion

To reduce the predicted structures to unique entries in the PCOD, originally the coordination sequences out to the 12^th^ shell at each crystallographically distinct T atom were compared.^6^ However, it is possible although rare for two structures with distinct topologies to have identical coordination sequences up to a given shell. So, for example RHO (Table SI1†) but not LTA (Table 3) has been retained in the PCOD, as they have the same coordination sequence.^6^ The ToposPro algorithm provides additional criteria to determine the overlap between the PCOD and IZA databases, and for that matter between PCOD and other structural databases. In particular, RHO and LTA are distinguished in ToposPro by their extended point symbols:^28^ [4.4.4.6.6.6] and [4.6.4.6.4.8_3_], respectively.

Three very recently discovered new zeolites, EMM-37,^18^ ECNU-21,^19^ and PST-30,^20^ also have frameworks that were predicted in the PCOD. Of these, PST-30 has a framework that was *a priori* designed from known building blocks of existing frameworks using rational design of a structure directing agent.^20^

It is interesting to note that of the 118 MOF topologies found in the PCOD, 46 are also in the IZA database. Conversely there are 11 MOF topologies present in the IZA database but not found in the PCOD. In addition, there are two interrupted MOF structures that are also found in the IZA database.

The ToposPro approach thus has enabled us to exhaustively and unequivocally identify existing zeolites, zeolite-like materials, and MOFs that have been predicted by the purely theoretical and unbiased methods used to generate the PCOD. This is very promising as it is an indication of the practical synthesizability of these hitherto predicted compounds. This in turn motivates the development and application of algorithms to further mine predicted structure databases for novel materials with desired or tailored properties.^24,33^ We have included the PCOD topologies into the ToposPro TTD Collection as a separate predicted zeolite database and provided a remote access to the database through our TopCryst service. One can use ToposPro to generate the underlying net for any zeolite-like framework and then check if the framework was already generated as a predicted zeolite.

Our analysis has not considered chirality. Only one of the two possible chiral forms for non-centrosymmetric structures is included in the PCOD database. The other chiral form is found by inversion.

## Conclusion

It has been known that a number of predicted zeolite structures in the PCOD database corresponded to existing zeolite materials in the IZA database.^6^ Using the ToposPro program we have further confirmed this and also found that newly discovered zeolites had been predicted by the PCOD. In addition we have found that other nanoporous materials such as inorganic zeolite-like compounds and MOFs have topologies that are present in the PCOD. This confirms that PCOD has a great potential for screening of novel nanoporous materials for selected applications and their eventual synthesis and use.

## Conflicts of interest

Michael W. Deem is a consultant for the petrochemical industry in the area of zeolites. This relationship did not affect the design or outcome of the present research.

## Supplementary Material

RA-010-D0RA01888K-s001
