# Supplementary material for: Nanoporous materials with predicted zeolite topologies
Source: RSC Adv. 2020 May 8;10(30):17760–7. doi: 10.1039/d0ra01888k (PMC9053620; doi:10.1039/d0ra01888k)
Supplement: RA-010-D0RA01888K-s001 [file RA-010-D0RA01888K-s001.pdf]

Electronic Supplementary Material (ESI) for RSC Advances.
This journal is © The Royal Society of Chemistry 2020

Table SI1: IZA zeolites that are present in the PCOD.

PCOD code	IZA code	PCOD code	IZA code	PCOD code	IZA code
PCOD8052570	ACO	PCOD8132964	EZT	PCOD8309058	POR
PCOD8077977	AEI	PCOD8307996	FAU	PCOD8318264	POS
PCOD8114334	AEL	PCOD8115812	FER	PCOD8124112	PWO
PCOD8249723	AEN	PCOD8323748	FRA	PCOD8133881	PWW
PCOD8322709	AFG	PCOD8000282	GIS	PCOD8306957	RHO
PCOD8324445	AFI	PCOD8077978	GME	PCOD8118223	RRO
PCOD8125830	AFN	PCOD8166073	GON	PCOD8048551	RTE
PCOD8077632	AFO	PCOD8158586	GOO	PCOD8058685	RTH
PCOD8068316	AFR	PCOD8094207	HEU	PCOD8107205	RUT
PCOD8323974	AFS	PCOD8236886	IFO	PCOD8187693	RWR
PCOD8330864	AFT	PCOD8038881	IFR	PCOD8295280	SAF
PCOD8321600	AFV	PCOD8306634	IFY	PCOD8309154	SAO
PCOD8321918	AFX	PCOD8316304	ISV	PCOD8085224	SAS
PCOD8054148	AFY	PCOD8082737	ITE	PCOD8095768	SAT
PCOD8052270	AHT	PCOD8129364	ITW	PCOD8308796	SAV
PCOD8308045	ANA	PCOD8060435	IWR	PCOD8320023	SBE
PCOD8248926	APC	PCOD8285528	IWV	PCOD8124791	SBN
PCOD8226985	APD	PCOD8011377	JBW	PCOD8328215	SBS

PCOD8115801	AST	PCOD8125027	JNT	PCOD8124190	SFE
PCOD8307029	ASV	PCOD8156657	JRY	PCOD8077517	SFF
PCOD8030519	ATO	PCOD8117232	JSN	PCOD8071784	SFH
PCOD8132873	ATS	PCOD8248916	JSW	PCOD8041875	SFN
PCOD8228636	ATT	PCOD8307701	KFI	PCOD8052725	SFO
PCOD8152280	ATV	PCOD8117704	LAU	PCOD8324260	SFW
PCOD8321605	AVL	PCOD8095118	LEV	PCOD8151609	SGT
PCOD8248929	AWO	PCOD8321851	LIO	PCOD8077983	SIV
PCOD8072989	AWW	PCOD8136440	LOS	PCOD8321332	SOD
PCOD8067826	BCT	PCOD8141540	LOV	PCOD8301730	SOR
PCOD8067418	BEC	PCOD8046159	LTJ	PCOD8325096	SSF
PCOD8169309	BIK	PCOD8327203	MAR	PCOD8118007	SSY
PCOD8076973	BOF	PCOD8323247	MAZ	PCOD8028960	STF
PCOD8298667	BOG	PCOD8324829	MEI	PCOD8186325	STI
PCOD8327297	BPH	PCOD8311069	MEL	PCOD8325990	STW
PCOD8045908	BRE	PCOD8331074	MEP	PCOD8105936	SVV
PCOD8001707	CAN	PCOD8306691	MER	PCOD8330642	SWY
PCOD8184706	CAS	PCOD8312053	MON	PCOD8276108	SZR
PCOD8184697	CDO	PCOD8115524	MOR	PCOD8238986	THO
PCOD8156936	CFI	PCOD8119854	MRT	PCOD8323760	TOL

PCOD8077973	CGS	PCOD8324148	MSO	PCOD8170391	TON
PCOD8068050	CHA	PCOD8304448	MTN	PCOD8320020	TSC
PCOD8087464	CON	PCOD8229816	MTT	PCOD8053433	UEI
PCOD8115528	DAC	PCOD8128662	MTW	PCOD8306851	UFI
PCOD8162585	DFT	PCOD8170877	MVY	PCOD8123039	UOE
PCOD8323956	DOH	PCOD8157946	NAT	PCOD8123038	UOS
PCOD8183569	DON	PCOD8285546	NES	PCOD8307096	UOZ
PCOD8321606	EAB	PCOD8283526	NON	PCOD8078892	USI
PCOD8308791	EDI	PCOD8052098	NSI	PCOD8302336	VET
PCOD8325727	EMT	PCOD8321330	OFF	PCOD8327509	VFI
PCOD8129041	EPI	PCOD8263729	OSI	PCOD8055190	YUG
PCOD8321602	ERI	PCOD8122541	OWE	PCOD8123580	ZON
PCOD8252730	ESV	PCOD8056793	PHI		
PCOD8322713	ETR	PCOD8168597	PON		
